# Psychological mechanisms linking a financial-literacy curriculum to scenario-based financial decision quality: a cluster randomized trial of financial self-efficacy and future-orientation in Chinese vocational students

**DOI:** 10.3389/fpsyg.2026.1906773

**Published:** 2026-08-10

**Authors:** Shaoran Yu, Zhao Zhang

**Affiliations:** 1 Department of Economics & Trade, Deyang Vocational College of Technology and Trade, Deyang, China; 2 School of Sports Training, Chengdu Sport University, Chengdu, China

**Keywords:** cluster randomized trial, consideration of future consequences, educational intervention, financial decision-making, financial self-efficacy, financial-literacy education, multiple mediation, vocational students

## Abstract

**Background:**

Financial-literacy education can change behavior, but the psychological mechanisms through which it works and how long its effects last remain underspecified, especially for underrepresented learners.

**Methods:**

In a cluster randomized trial, four first-year classes (*N* = 320) at a Chinese vocational college were allocated at the class level to a four-week, eight-session financial-literacy curriculum or an active control. Financial knowledge, financial self-efficacy, future-orientation (CFC), risk and debt attitudes, and a scenario-based behavioral decision-quality index were measured at baseline, immediately afterward, and 6 months later.

**Results:**

Under cluster-respecting inference—the appropriate basis for causal claims with class-level randomization—no outcome attained conventional significance: all enumerated wild-cluster-bootstrap *p* values sit at the structural floor of 0.125 imposed by four clusters, and exact randomization inference is likewise bounded (*p* = 0.333). As a cluster randomized trial, the study therefore cannot reject the null at the unit of randomization. Individual-level difference-in-differences estimates (knowledge *d* = 0.37; self-efficacy *d* = 0.51; decision quality *d* = 0.48) are reported as effect-size estimates requiring confirmation in adequately clustered trials. In exploratory, individual-level mediation analysis, financial self-efficacy and future-orientation (CFC) appeared as parallel partial mediators, jointly accounting for 41% of the effect on decision quality; sensitivity analysis shows these indirect effects would be nullified by modest mediator–outcome confounding. Knowledge estimates faded by 6 months, whereas self-efficacy showed a directional but non-significant trend toward persistence.

**Conclusion:**

The findings provide effect-size estimates and mechanism hypotheses—capacity beliefs and future-orientation as parallel pathways—for adequately clustered confirmatory trials of financial-literacy education in this underserved population.

## Highlights

- A four-week financial-literacy curriculum was evaluated in a four-class cluster randomized trial (*N* = 320) of Chinese vocational students.
- Individual-level estimates suggested gains in financial knowledge (*d* = 0.37), self-efficacy (*d* = 0.51), and scenario-based decision quality (*d* = 0.48); none attains significance under cluster-respecting inference.
- In exploratory, individual-level mediation analysis, financial self-efficacy and future-orientation appeared as parallel mediators, jointly accounting for 41% of the effect on decision quality.
- Knowledge gains faded by 6 months, whereas self-efficacy showed a directional but non-significant trend toward persistence.
- With only four clusters, cluster-respecting inference is the primary basis for causal claims; all immediate effects sit at the structural floor of the enumerated wild cluster bootstrap (minimum attainable *p* = 0.125).

## 1 Introduction

How a classroom curriculum changes what learners believe about their own capabilities and how those beliefs translate into decision-making is a core question of educational psychology (Bandura, 1997). Financial literacy, the ability to understand and apply economic concepts to personal financial decisions, has emerged as a critical determinant of individual welfare and broader macroeconomic outcomes (Lusardi and Mitchell, 2014). A growing meta-analytic literature has converged on the conclusion that financial education programs, when delivered with adequate dosage and rigor, produce causal improvements in both financial knowledge and downstream behaviors. The most comprehensive synthesis to date, by Kaiser et al. (2022), drew on 76 randomized experiments across 33 countries and over 160,000 participants and found average treatment effects three to five times larger than earlier meta-analytic estimates. Their analysis confirmed that financial education is “an effective tool for increasing financial knowledge and improving financial behavior,” but also revealed substantial heterogeneity attributable to context, dosage, and population characteristics.

Despite this maturing evidence base, three substantive gaps constrain its practical relevance, particularly for emerging-economy policy.

### 1.1 Gap 1: Chinese vocational students are underrepresented

Existing rigorous RCTs predominantly sample U.S. or European secondary or tertiary general students (Bover et al., 2026; Iterbeke et al., 2020). Chinese students—whose financial knowledge lags international peers (only 33% reach high proficiency versus 42% in the OECD/INFE comparison group), and whose overall financial-literacy score of 13.1 on the 20-point OECD/INFE scale falls below the OECD benchmark of 14 despite exceeding the 17-country average of 11.7 (Tan et al., 2024)—are rarely included, and Chinese vocational students remain virtually absent. Yet vocational students constitute over 16 million Chinese tertiary enrollees and frequently come from lower-income, less educated households—a context where randomized evidence from comparable lower- and middle-income settings indicates that financial-education interventions can produce meaningful gains in financial knowledge and behavior (Doi et al., 2014). Given the documented heterogeneity of financial-education effects across populations (Kaiser and Menkhoff, 2017) and the policy salience of reaching an underserved group, this population is both methodologically valuable and policy-relevant.

### 1.2 Gap 2: psychological mechanisms investigated in isolation

How does financial education translate into financial decision-making? Two psychological pathways have received empirical support, but in separate literatures. First, financial self-efficacy, an individual’s perceived capacity to manage personal finances, has been shown to mediate the relationship between financial knowledge and behavior (Rothwell et al., 2016), drawing on Bandura’s (1997) self-efficacy theory. Educational interventions that strengthen self-efficacy beliefs are theorized to increase the probability that newly acquired knowledge translates into action (Lown, 2011). Second, economic preferences, particularly time preference (intertemporal discounting) and risk attitude, have been demonstrated to shift in response to a randomized financial-literacy intervention (Sutter et al., 2020), with preference change in turn predicting field financial behavior. Behavioral economics theory predicts that financial literacy should specifically reduce present bias and modulate excessive risk-taking by enhancing future-orientation and risk awareness.

To our knowledge, no study has integrated these two mechanisms within a single RCT, leaving open whether they operate as parallel pathways, substitute for one another, or interact. Such integration is essential for designing interventions that target the full causal chain rather than a single proxy. The mediator-isolation problem also raises concerns about effect size attribution: studies estimating “the” mediation effect of either single pathway may overstate the unique contribution of the studied mediator if it is correlated with unmeasured pathways operating in parallel.

### 1.3 Gap 3: limited six-month follow-up evidence

Kaiser et al. (2022) noted that long-term follow-up evidence remains relatively sparse, with outcomes measured a median of roughly 6 months after treatment and few studies tracking effects at 1 year or longer. The retention of treatment effects at 6 months is particularly relevant for educational policy because brief curricula are cheap to scale but valuable only insofar as their effects endure. Bover et al. (2026) provide a rigorous randomized demonstration of short-run gains among Spanish high-school students, and the few studies with longer horizons (e.g., Frisancho, 2023, in Peru; Bruhn et al., 2016, in Brazil) document both immediate gains and their partial attenuation over time, but comparable evidence in Chinese vocational settings is absent. Moreover, the differential decay rates of cognitive vs. psychological gains—e.g., whether self-efficacy or knowledge dissipates first—remains an open question with direct curricular design implications.

### 1.4 The present study

We address these three gaps by conducting a cluster-randomized controlled trial of a four-week financial literacy curriculum in a Chinese vocational college, with three measurement waves (baseline, immediate post-intervention, six-month follow-up) and a multiple-mediator design that simultaneously tests financial self-efficacy and future-orientation (CFC) as parallel pathways from education to scenario-based decision-quality outcomes. Our contribution thus lies in: (1) populational extension, to our knowledge the first rigorous RCT of financial literacy education in Chinese vocational tertiary students; (2) mechanistic integration, the first parallel multiple-mediator decomposition of self-efficacy and economic-preference pathways within a single design; and (3) temporal coverage, a six-month follow-up addressing the persistence question. Throughout, financial literacy serves as the curricular domain; the focal contribution concerns the psychological mechanisms, capacity beliefs and future-orientation, through which classroom instruction changes learner decision-making. Given the small number of randomized clusters, the study is positioned as providing effect-size estimates and mechanism hypotheses for adequately clustered confirmatory trials rather than definitive causal demonstration.

## 2 Theoretical framework and hypotheses

We adopt a multi-mechanism extension of the financial education–behavior framework (Lusardi and Mitchell, 2014), integrating Bandura’s (1997) self-efficacy theory and behavioral economics’ theory of intertemporal choice (Frederick et al., 2002). In our model, a curricular intervention operates through (a) a cognitive pathway, directly increasing financial knowledge and (b) two parallel psychological pathways: financial self-efficacy and the consideration of future consequences (CFC; Strathman et al., 1994). The two psychological pathways are hypothesized to jointly mediate the effect of education on scenario-based financial decision quality. We additionally examine effects on risk attitude (DOSPERT; Weber et al., 2002) and debt attitude (DAS; Davies and Lea, 1995) as theoretically relevant secondary outcomes that the curriculum addresses but for which we expect smaller effect sizes given the brief intervention dosage.

Bandura’s (1997) social-cognitive theory identifies four sources of efficacy beliefs (mastery experience, vicarious experience, verbal persuasion, and physiological states), of which mastery experience is the most powerful. The curriculum operationalizes this source directly: Week 4 requires each student to construct a personal six-month financial plan, a graded mastery task designed to convert newly acquired knowledge into a concrete experience of financial competence. Self-efficacy theory therefore predicts not only an immediate treatment effect on efficacy beliefs (H2) but also greater durability of that effect relative to declarative knowledge, because perceived competence is self-reinforcing through subsequent behavioral feedback (Bandura, 1997).

Consideration of future consequences is conceptualized as a relatively stable individual difference (Strathman et al., 1994), yet it is not fixed: intertemporal-choice instruction makes distant outcomes cognitively concrete (compound-interest projections, for example, render the future self’s budget visible), and randomized financial education has been shown to shift measured time preferences through precisely this channel (Sutter et al., 2020). We therefore treat CFC as a malleable proximal target of instruction rather than a fixed trait, while measuring it with an instrument validated as an individual-difference scale. CFC should nonetheless be distinguished from time preference in the economic sense: CFC is a self-reported psychological disposition toward weighing distant consequences, whereas time preference in Sutter et al. (2020) is estimated from incentivized intertemporal choices. The two are related but not interchangeable, and throughout this article we use future-orientation (CFC) to refer to our measure, reserving time preference for the incentivized parameter.

Five hypotheses:

*H1*. Treatment exceeds Control on financial literacy at T2 and T3.

*H2*. Treatment exceeds Control on financial self-efficacy at T2 and T3.

*H3*. Treatment shows greater future-orientation (CFC), more measured risk attitude (DOSPERT), and more cautious debt attitude (DAS) at T2 and T3.

*H4*. Treatment exceeds Control on a behavioral decision-quality index at T2 and T3.

*H5 (exploratory, hypothesis-generating)*. Financial self-efficacy and CFC jointly mediate the effect of treatment on scenario-based decision quality (parallel multiple mediator model). Because mediators and outcome are measured concurrently and clustering cannot be modeled with four clusters, H5 is examined as an exploratory, correlational decomposition rather than a confirmatory causal claim.

## 3 Materials and methods

### 3.1 Design

This was a two-arm, parallel-group, cluster randomized controlled trial with three measurement waves. Class-level (rather than individual-level) randomization was chosen to (a) prevent within-class spillover that would bias treatment estimates downward, and (b) align with the natural delivery unit of the intervention (a class-based course). All procedures were reviewed and approved by the Institutional Review Board of Chengdu Sport University (approval number CDSUIRB202504; approval date 29 September 2025) and conformed to the Declaration of Helsinki principles.

**Figure 1:**
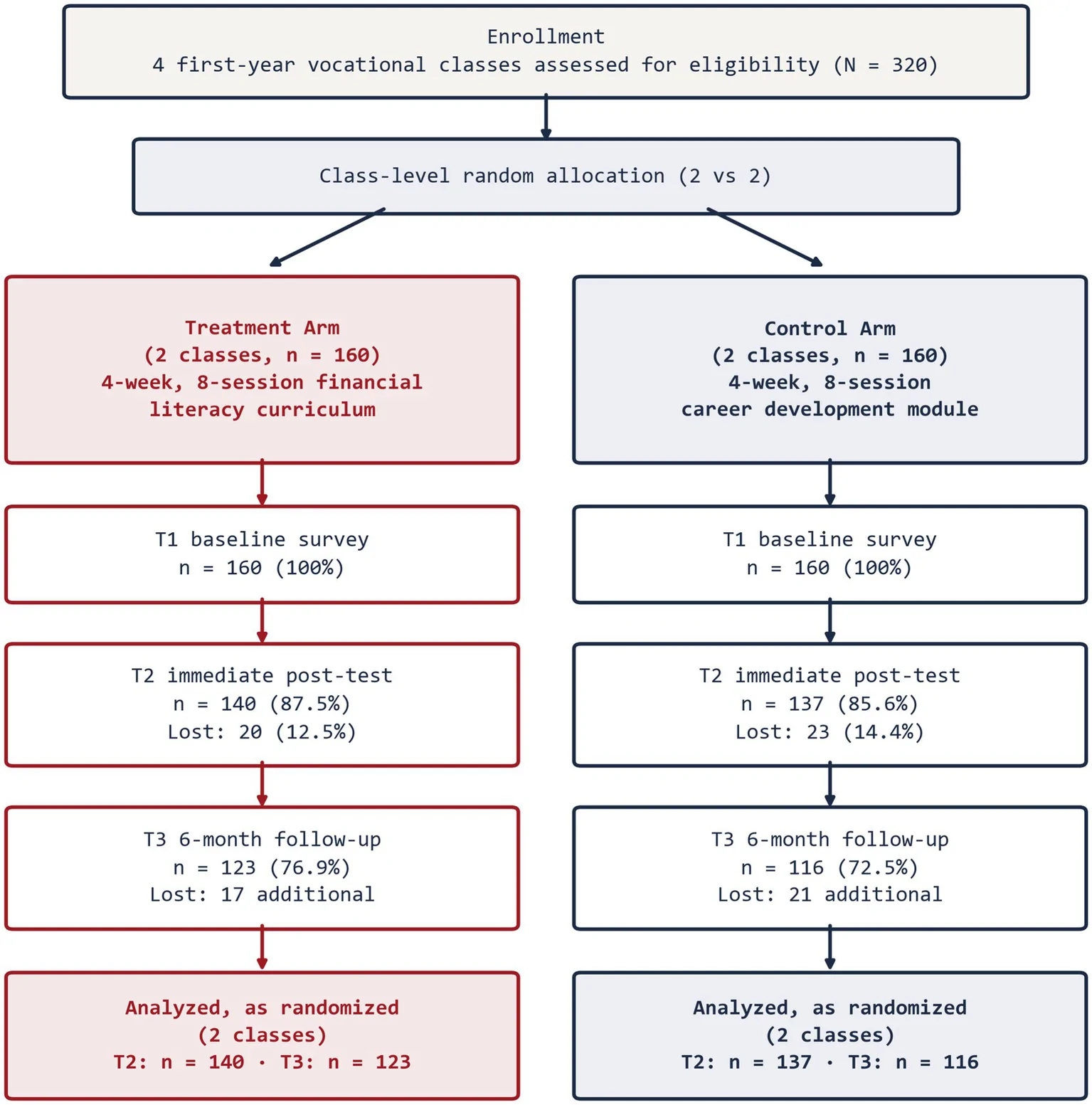
CONSORT flow diagram of participant allocation and retention across three measurement waves.

The CONSORT diagram (Figure 1) summarizes participant flow.

### 3.2 Participants and setting

Participants were *N* = 320 first-year students (*M*_age_ = 18.40 years, *SD* = 0.70) enrolled in non-finance majors at Deyang Vocational College of Technology and Trade, Deyang, China. We sampled non-finance majors specifically to avoid confounding from prior coursework. Four intact classes (≈80 students each) were randomly assigned: classes 1 and 2 to the treatment arm (*n* = 160) and classes 3 and 4 to the active control arm (*n* = 160).

All four classes were identified and enrolled, and baseline (T1) assessment was completed, before random assignment took place; allocation was therefore structurally concealed from participants, the instructor, and class advisers at enrollment. The allocation sequence was generated by an investigator blinded to class composition and with no contact with the classes, using a web-based randomization tool, and was communicated to the principal investigator only after baseline completion. Because clusters rather than individuals were randomized, no individual-level concealment mechanism applied, and post-allocation blinding of participants and instructor was not possible for an educational intervention of this type. Power analysis (G*Power 3.1; *α* = 0.05, two-tailed; *power* = 0.80; expected *d* = 0.30 informed by Kaiser et al., 2022) indicated approximately 176 participants per arm for an unadjusted two-group comparison; because the planned difference-in-differences estimator incorporates baseline measurements, which substantially reduces the variance of the treatment estimate, and in view of the cluster design effect (ICC ≈ 0.05) and projected attrition, a target enrollment of 300–400 was set. This individual-level calculation does not bound power for cluster-level inference: with only four randomized clusters, the trial is necessarily underpowered to detect effects at the unit of randomization, as the wild cluster bootstrap results in Section 4 make explicit. A cluster-based power analysis was not conducted prospectively because the number of clusters was fixed by feasibility: four intact classes were available. Retrospectively, with *J* = 4 the cluster-level comparison has two degrees of freedom, and the minimum detectable effect size at 80% power is approximately *d* = 1.33 at the assumed ICC of 0.05 (*d* = 0.96 at ICC = 0.02; *d* = 0.80 at ICC = 0.01; observed baseline ICCs ranged from 0.00 to 0.04). Effects of realistic magnitude were therefore undetectable at the cluster level by design.

Inclusion criteria: enrolled first-year vocational student, voluntary informed consent, no prior systematic financial literacy training (self-reported). Exclusion criteria: refusal to consent, inability to complete the four-week curriculum block.

### 3.3 Intervention

*Treatment arm*. Students received an eight-session, four-week curriculum (90 min/session = 12 h total) covering: Week 1, financial fundamentals (compound interest, inflation, diversification—Lusardi and Mitchell (2014) “Big Three”); Week 2, risk and decision-making (DOSPERT five-domain framework, prospect theory, risk perception vs. preference distinction); Week 3, debt and credit (effective annual interest rate computation, mental accounting, credit reports); and Week 4, intertemporal choice and self-efficacy strengthening (hyperbolic discounting, behavioral biases identification, personal six-month financial plan creation). Each session combined didactic lecture (45 min), case-based group discussion (30 min), and individual exercise (15 min). Detailed session-level lesson plans are available in Supplementary file S1.

*Active control arm*. Students received an equivalent eight-session block of the regular Career Development module (also 12 h), with no financial-literacy content. The active-control design controls for non-specific exposure effects (e.g., novelty, instructor attention). Following IRB equity provisions, control participants received free access to the full intervention materials after completion of the six-month follow-up.

### 3.4 Measures

All scales were administered in Chinese at three time points: T1 (1 week pre-intervention), T2 (1–3 days post-intervention), T3 (6 months post-intervention).

- Financial Literacy Scale (FLS): 5-item scale adapted from Lusardi and Mitchell (2014), assessing core financial concepts (compound interest, inflation, diversification, interest-rate sensitivity, credit-card mechanics). Each correct answer = 1 point; range 0–5.
- Financial Self-Efficacy Scale (FSES): 6-item Likert scale (Lown, 2011), measuring confidence in managing personal finances. Items reverse-coded so that higher scores indicate higher self-efficacy. Responses were made on a 4-point scale (1–4) and scored as the item mean (range 1–4). The Chinese translation underwent translate-back-translate procedures with a pilot sample (*N* = 40) yielding internal consistency Cronbach’s *α* = 0.81; formal psychometric validation in a larger Chinese vocational population remains a needed future step (see Limitations).
- Domain-Specific Risk-Taking — Financial Subscale (DOSPERT): 6-item Likert scale (Weber et al., 2002), assessing financial risk-taking propensity. Responses were made on a 7-point scale (1–7) and scored as the item mean (range 1–7), with higher scores indicating greater risk-taking propensity.
- Debt Attitude Scale (DAS): 8-item Likert scale (Davies and Lea, 1995; adapted from Norvilitis et al., 2003), measuring tolerance for and engagement with debt. Responses were made on a 5-point scale (1–5) and scored as the item mean (range 1–5).
- Consideration of Future Consequences (CFC): 7-item Likert scale (Strathman et al., 1994), assessing tendency to consider distant outcomes when making decisions. Responses were made on a 5-point scale (1–5) and scored as the item mean (range 1–5).
- Behavioral Decision Quality Index (BDQ): a scenario-based index of financial decision-making tendencies, computed as a composite score across 5 simulated financial decision scenarios (intertemporal choice, risk allocation, debt evaluation, consumption decision, planning behaviors), each scored 0 or 1 against an *a priori* rubric. The composite is the mean of the five scenarios; range 0–1. Convergent validity at baseline was supported by significant positive correlations of BDQ with self-reported habitual planning behaviors (budgeting *r* = 0.35; expense tracking *r* = 0.24; goal-setting *r* = 0.33; comparison shopping *r* = 0.40; mean *r* = 0.33, all *p* < 0.001), indicating that BDQ taps an enduring decision-quality construct rather than transient momentary judgment. Because the scenarios are simulated and rubric-scored, BDQ measures decision-making tendencies rather than observed field behavior, and we interpret it accordingly throughout; the planning-behavior anchors are themselves self-reports and cannot elevate the measure to observed behavior.
- Manipulation Check (T2 only; administered in both arms, each rating its own curriculum): 5-item Likert assessing curriculum quality and self-perceived utility. Predefined threshold: mean ≥ 4.0 of 5.0 indicates successful implementation.

Cronbach’s *α* for all scales in our sample exceeded 0.70 (DAS = 0.77; FSES = 0.81; CFC = 0.73; DOSPERT = 0.76); FLS reliability assessed via KR-20 = 0.68. The lower FLS reliability reflects the intentionally heterogeneous item-difficulty distribution—covering compound interest, inflation, diversification, interest-rate sensitivity, and credit-card mechanics—rather than measurement noise. Nevertheless, KR-20 = 0.68 remains low; it both attenuates observed effects and inflates their sampling variability, and we flag it as a limitation specific to the literacy outcome (Section 5.3). The full questionnaire is available in Supplementary file S2.

### 3.5 Procedure

Students in both arms completed an online questionnaire at T1, attended the eight-session curriculum block, completed T2 within 1–3 days of the final session, and completed T3 6 months later. Recruitment and the baseline (T1) assessment took place in October 2025, and the six-month follow-up (T3) was completed in May 2026. To mitigate teacher–pupil power asymmetry that could bias responses, survey administration was conducted by a research assistant—not the instructor. An anonymous matching code (last 4 digits of student ID + birth month) was used to link responses across waves; no personally identifying information was collected. Informed consent was obtained from all participants involved in the study, at both the cluster (college/class) and the individual student level. Most participants were adults (18 years or older); for the subset of first-year students who were 17 years old at enrollment, written informed consent was additionally obtained from a parent or legal guardian, and these students themselves provided written assent.

Data availability statement: The de-identified three-wave dataset and complete analysis code supporting the conclusions of this article are publicly available in the Open Science Framework repository at https://osf.io/ep7v4 (DOI: 10.17605/OSF.IO/EP7V4).

Instructor consistency: Both treatment and active-control sessions were delivered by the same instructor (the PI), who delivered the active-control content with comparable enthusiasm, time investment, and instructional structure. While this design avoids confounding between intervention content and instructor identity, it precludes instructor blinding to study hypothesis—a limitation we discuss in Section 5.3.

Manipulation check: At T2, both arms completed a 5-item Likert evaluation of their respective curriculum (the Treatment arm rated the financial literacy curriculum; the Control arm rated the Career Development module). Differences in evaluation between arms were tested to verify that any treatment effects could not be solely attributed to differential perceived course quality.

### 3.6 Statistical analysis

Primary specification. Because the trial included only 4 clusters (*J* = 4) of approximately 80 students each, conventional cluster random-effects estimation is unreliable in this setting, because the second-level (cluster) standard errors are biased when the number of clusters is small (Maas and Hox, 2005, who report biased level-two standard errors with 50 or fewer groups). We therefore adopted the following inference hierarchy, in which cluster-respecting inference constitutes the primary basis for causal claims at the unit of randomization.

Individual-level estimation used OLS difference-in-differences (DID) regression with class fixed effects and HC3 heteroskedasticity-robust standard errors:

$$\begin{array}{l} Y_{\mathrm{ict}}=\beta_{0}+\beta_{1}{\text{Post}}_{t}+\beta_{2}{\left(\text{Treatment}\times \text{Post}\right)}_{\mathrm{ct}}+\\ \gamma^{\prime }C_{c}+\delta {\text{Gender}}_{i}+\varepsilon_{\mathrm{ict}} \end{array}$$

where $C_{c}$ is a vector of class fixed effects (absorbing time-invariant class differences and identifying the treatment indicator from the within-class baseline-vs-postintervention difference). *β*₂ is the treatment effect of interest. Gender is included as an individual-level control; the one baseline covariate showing marginal imbalance, parental education, was added in a robustness check (reported in Section 4.1) that left all conclusions unchanged. HC3 robust standard errors guard against arbitrary heteroskedasticity at the individual level. Because treatment was assigned at the class level, the nominal precision of these individual-level estimates rests on an independence assumption that the design does not license; they are therefore reported as effect-size estimates, not as the basis for causal claims.

Cluster-respecting inference: wild cluster bootstrap (WCB). Given only 4 clusters, individual-level inference understates clustering-induced variance. We therefore base causal inference at the unit of randomization on a wild cluster bootstrap-*t* procedure (Cameron et al., 2008) with enumerated Rademacher weights across all 2^4^ = 16 sign combinations; with only four clusters this enumeration is exhaustive rather than sampled. Webb (2014) shows that with few clusters the two-point Rademacher wild bootstrap admits only a small, fixed number of distinct draws (here, 16), which is the source of the discreteness reported below. Two of the sixteen draws (the identity and its full sign reversal) reproduce the observed |*t*| exactly and are always counted, so *p* values follow the exact-test convention *p* = *k*/16, where *k* is the number of the 16 enumerated draws whose |t*| is at least as large as the observed |*t*|; the structural floor is therefore 2/16 = 0.125; consequently, conventional 0.05 significance is unattainable at the cluster level by design. Both individual-level and cluster-respecting *p* values are reported in Table 1; the latter constitute the primary basis for causal claims.

**Table 1:** Difference-in-differences treatment effects at T2 and T3—wild cluster bootstrap (primary cluster-respecting inference) and OLS with class fixed effects and HC3 robust SE (individual-level effect-size estimates).

Outcome	Wave	*β* (HC3 SE)	*p* (robust)	*p* (WCB)	*d*
FLS (financial literacy)	T2	*0.504 (0.22)*	*0.023*	0.125	0.37
FLS	T3	0.245 (0.23)	0.288	0.250	0.18
FSES (self-efficacy)	T2	*0.442 (0.14)*	*0.001*	0.125	0.51
FSES	T3	0.242 (0.14)	0.093	0.125	0.28
CFC (future-orientation)	T2	0.300 (0.17)	0.084	0.125	0.30
CFC	T3	0.101 (0.18)	0.572	0.250	0.10
DOSPERT (risk)	T2	−0.302 (0.26)	0.242	0.125	−0.19
DOSPERT	T3	−0.152 (0.27)	0.574	0.125	−0.10
DAS (debt attitude)	T2	−0.258 (0.18)	0.141	0.125	−0.24
DAS	T3	−0.189 (0.18)	0.302	0.375	−0.18
BDQ (decision quality)	T2	*0.125 (0.04)*	*0.002*	0.125	0.48
BDQ	T3	0.037 (0.05)	0.413	0.750	0.14

WCB = wild cluster bootstrap-t (Cameron et al., 2008) with enumerated Rademacher weights across 2^4^ = 16 sign combinations; *p* = *k*/16, where k is the number of enumerated draws whose |t*| is at least as large as the observed |*t*| (exact-test convention; the identity and sign-reversed draws always attain the observed |*t*|), structural floor 2/16 = 0.125. WCB constitutes the primary basis for causal inference at the unit of randomization; the OLS class-fixed-effects HC3 column provides individual-level effect-size estimates whose nominal precision assumes an independence the design does not license. *β* = treatment-by-post interaction coefficient. *d* = *β*/pooled baseline SD. Bold highlights *p* < 0.05 under individual-level HC3 inference only; this does not constitute cluster-level significance. All immediate (T2) effects sit at the WCB structural floor; no outcome attains conventional cluster-level significance. Italicized values indicate *p* < 0.05 under individual-level HC3 inference only and do not constitute cluster-level significance.

Exploratory mediation analysis (H5) used a parallel-mediator model with FSES_T2 and CFC_T2 as parallel mediators of the Treatment → BDQ_T2 path, with each mediator model controlling for the mediator’s own baseline (FSES_T1 or CFC_T1) and gender, and the outcome model controlling for the baseline outcome (BDQ_T1) and gender. Indirect effects were estimated via bootstrap (2,000 resamples) and considered significant if their 95% percentile confidence intervals excluded zero. We acknowledge that mediator (M) and outcome (Y) are measured concurrently at T2, which limits the strength of causal interpretation: the parallel-mediator pattern is consistent with the hypothesized mechanism but does not rule out reverse M ← Y paths. Moreover, random assignment identifies only the treatment→mediator (a) paths; the mediator → outcome (b) paths remain observational and vulnerable to unmeasured confounding Imai et al. (2010a). Section 5.3 elaborates on this concern. The analysis followed Hayes (2017) PROCESS Model 4 logic, implemented in Python statsmodels and verified against Stata paramed. Because a cluster bootstrap is degenerate with four clusters, clustering cannot be modeled in the indirect-effect confidence intervals; instead, each path of the mediator model was re-estimated under the enumerated wild cluster bootstrap described above, and we additionally conducted the parametric sensitivity analysis of Imai et al. (2010b), in which the sensitivity parameter *ρ* is the correlation between the errors of the mediator and outcome equations and the average causal mediation effect is nullified when ρ equals the observed residual correlation. Given these constraints and the concurrent measurement of mediators and outcome, the mediation analysis is reported as exploratory and hypothesis-generating.

Sensitivity analyses: (a) analysis of all retained participants as randomized versus per-protocol (≥6/8 sessions); (b) inverse probability weighting (IPW) for differential attrition; (c) winsorization at the 1st/99th percentiles; (d) ANCOVA replacing DID; (e) adding parental education as a covariate. As a further cluster-respecting check, we conducted exact randomization inference by enumerating all C(4,2) = 6 possible assignments of the four classes to the two arms and recomputing the treatment×post coefficient under each; the two-sided randomization-inference *p* value is the proportion of assignments yielding |b| at least as large as observed. Heterogeneity by gender, parental education, family income, and baseline literacy quartile was examined exploratorily and labeled as such in Section 4.7. To assess common-method variance, a Harman single-factor test was conducted on all 27 self-report Likert items (DOSPERT, DAS, CFC, FSES).

Analytic transparency. The shift from cluster random effects to OLS-with-class-FE + WCB is reported here as a methodological refinement responding to known small-J inference issues, and is accompanied by both estimation strategies for full transparency.

All analyses used Python 3.14 (statsmodels 0.14, NumPy 2.3, pandas 2.3) with verification of key results in Stata 17.0. The de-identified three-wave dataset and the full analysis code are publicly available at https://osf.io/ep7v4 (DOI: 10.17605/OSF.IO/EP7V4).

## 4 Results

### 4.1 Descriptive statistics and baseline equivalence

Baseline characteristics by arm are summarized in Table 2. Treatment and control groups did not differ significantly on any of the six primary outcome measures or on gender or family income (all *p*s > 0.12). A marginal imbalance in parental education (*p* = 0.032) was observed; adding it as a covariate in a robustness check left every treatment estimate essentially unchanged (Δ*b* < 0.01; Section 3.6), so we report the gender-adjusted models as primary. The two arms were otherwise considered adequately matched at baseline. A Harman single-factor test on the 27 self-report Likert items at T2 showed the first unrotated factor accounting for 22.0% of total variance (21.2% at baseline; four factors with eigenvalues above 1), well below the conventional 50% threshold, suggesting that common-method variance is unlikely to dominate the self-report measures.

**Table 2:** Baseline descriptives and equivalence test by arm (*N* = 320).

Variable	Treatment *M* (*SD*)	Control *M* (*SD*)	*t*	*p*
Financial literacy (FLS, 0–5)	1.74 (1.41)	1.77 (1.31)	−0.21	0.837
Financial self-efficacy (FSES, 1–4)	2.48 (0.90)	2.53 (0.82)	−0.52	0.604
Future-orientation (CFC, 1–5)	3.03 (1.07)	2.96 (0.97)	0.56	0.578
Risk attitude (DOSPERT, 1–7)	4.10 (1.59)	3.89 (1.52)	1.22	0.225
Debt attitude (DAS, 1–5)	2.97 (1.10)	3.03 (1.03)	−0.54	0.591
Behavioral decision quality (BDQ, 0–1)	0.43 (0.27)	0.42 (0.26)	0.44	0.663
Gender (1 = male, 2 = female)	1.69	1.61	1.52	0.129
Parental education (1–5)	2.06 (1.00)	2.29 (0.97)	−2.15	*0.032*
Family income (1–6)	2.36 (1.07)	2.36 (1.06)	0.00	1.000

Likert scales reverse-coded as needed before averaging.

### 4.2 Attrition

Of the original *N* = 320, 277 (86.6%) completed T2 and 239 (74.7%) completed T3. Logistic regression of T2 retention on treatment, parental education, family income, and gender showed no significant effect of treatment assignment (*p* = 0.430), suggesting attrition was not selective with respect to arm. A similar non-selective pattern held at T3. At the cluster level, retention was 91.2%, 83.8%, 86.2%, and 85.0% for classes 1–4 at T2, and 80.0%, 73.8%, 76.2%, and 68.8% at T3; retention did not differ significantly across classes (T2: *χ*^2^(3) = 2.23, *p* = 0.526; T3: *χ*^2^(3) = 2.83, *p* = 0.419). We note, however, that with four clusters individual-level inverse probability weighting cannot repair class-level selective attrition, so follow-up estimates additionally assume that within-class attrition mechanisms are captured by observed covariates. Treatment-effect models were accordingly estimated on the *n* = 277 participants retained at T2 and the *n* = 239 retained at T3 (all retained participants analyzed as randomized); the mediation model (Section 4.6) used the *n* = 277 T2 sample. No harms or unintended adverse effects related to the intervention were reported.

### 4.3 Manipulation check

Treatment-arm participants rated the financial literacy curriculum favorably (*M* = 4.16 of 5, *SD* = 0.28), exceeding the predefined threshold of 4.0 for “successful implementation.” Active-control participants rated the Career Development module also favorably (*M* = 3.78, *SD* = 0.31). The two arms differed by 0.38 points on the perceived-quality measure. Both exceeded the implementation threshold, but this difference is equally consistent with differential engagement or demand effects as with a targeted-content advantage, and we do not interpret it as evidence against expectancy confounds (Section 5.3).

### 4.4 Treatment effects at T2 (immediate post-intervention)

Table 1 presents DID estimates. Under individual-level inference, treatment increased financial literacy (*β* = 0.50, *SE* = 0.22, *p* = 0.023, *d* = 0.37), financial self-efficacy (*β* = 0.44, *SE* = 0.14, *p* = 0.001, *d* = 0.51), and behavioral decision quality (*β* = 0.13, *SE* = 0.04, *p* = 0.002, *d* = 0.48). Future-orientation (CFC) showed a marginal improvement (*p* = 0.084, *d* = 0.30). Risk attitude (DOSPERT) and debt attitude (DAS) shifted in the predicted directions (toward more cautious responses) but did not reach conventional significance, consistent with the relatively brief intervention dosage. Because six outcomes were each tested at two waves (12 primary tests), only self-efficacy (*p* = 0.001) and behavioral decision quality (*p* = 0.002) survive a Bonferroni-corrected threshold (0.05/12 ≈ 0.004); the financial-literacy effect (*p* = 0.023) should accordingly be interpreted with caution. Under the cluster-respecting wild cluster bootstrap—the primary basis for causal claims in this design—every immediate effect sits at the structural floor (all WCB *p* = 0.125; Table 1), so none attains conventional significance at the unit of randomization.

**Figure 2:**
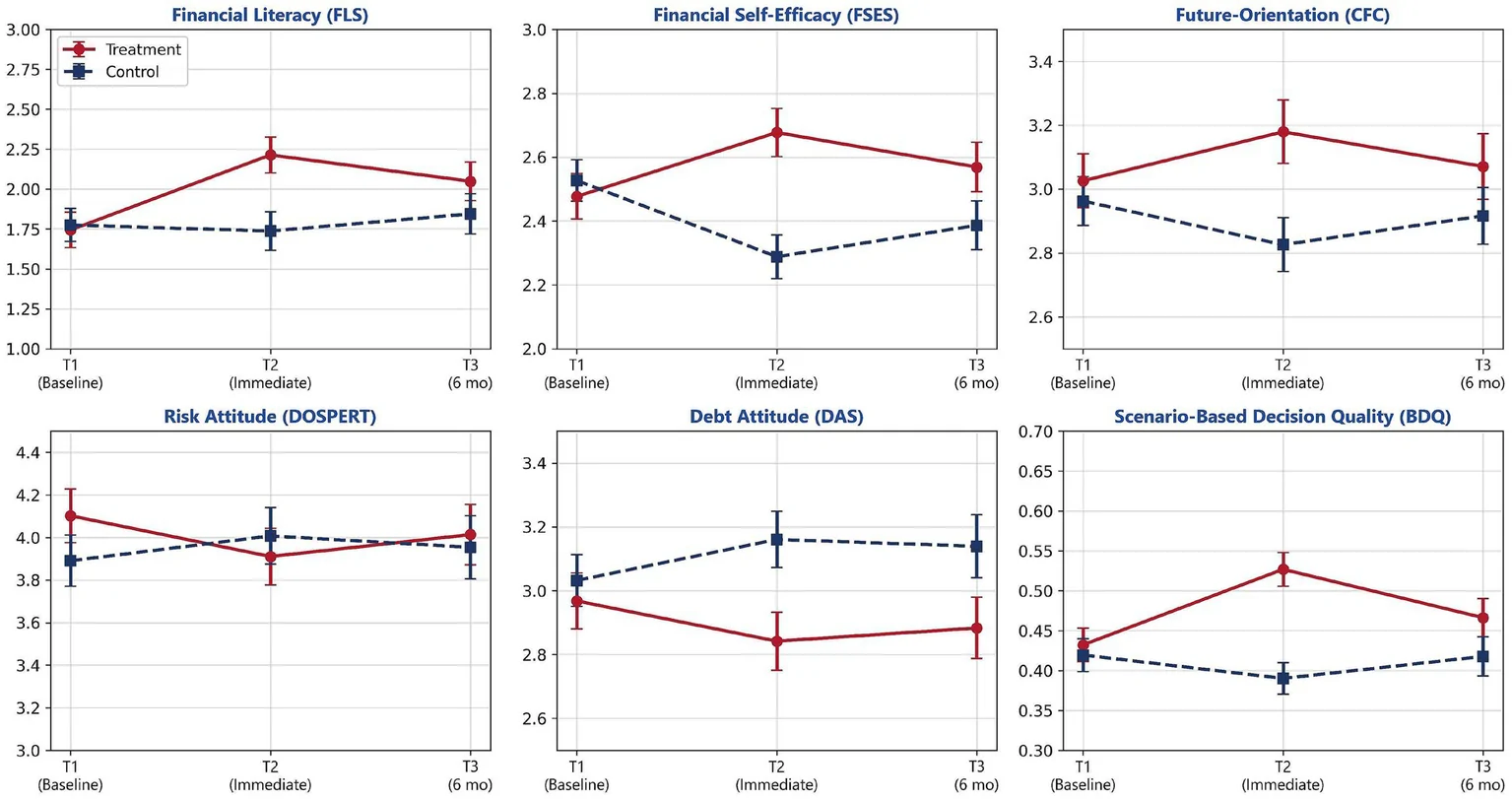
Three-wave trajectories of all six outcome variables by treatment arm (mean ± SEM).

Three-wave trajectories for all outcomes are displayed in Figure 2. The immediate elevation followed by partial decay is visually consistent with the meta-analytic pattern reported by Kaiser et al. (2022).

### 4.5 Treatment effects at T3 (6-month follow-up)

At T3, effects on financial literacy and scenario-based decision quality fully attenuated to non-significance under both HC3 and WCB inference (FLS: HC3 *p* = 0.288, WCB *p* = 0.250; BDQ: HC3 *p* = 0.413, WCB *p* = 0.750), although point estimates remained directionally consistent with the immediate findings. The effect on financial self-efficacy showed a directional but non-significant trend under HC3 (*β* = 0.24, *p* = 0.093, *d* = 0.28) and did not approach significance under WCB (*p* = 0.125). Descriptively, knowledge estimates fully attenuated within 6 months while the self-efficacy and future-orientation point estimates partially retained their direction; we treat this durability contrast as a hypothesis for longitudinal replication rather than as an established differential-decay finding. This reading is compatible with the meta-analytic findings of Kaiser et al. (2022), which found short-run knowledge gains somewhat smaller at horizons of 6 months or more, though with the longer-run evidence judged inconclusive given few long-horizon studies.

### 4.6 Exploratory mediation analysis (H5)

Table 3 reports the bootstrap multiple-mediator analysis; consistent with Section 3.6, these results are exploratory and individual-level. Under individual-level inference, both mediators showed indirect effects whose bootstrap CIs excluded zero: indirect via FSES = 0.038, 95% CI [0.022, 0.056]; indirect via CFC = 0.018, 95% CI [0.008, 0.030]. The total indirect effect was 0.056, 95% CI [0.037, 0.078], representing 41% of the total treatment effect (0.135). The direct effect remained significant (*c*′ = 0.079, 95% CI [0.026, 0.129]), indicating partial mediation: financial self-efficacy and future-orientation jointly account for a substantial—but not complete—portion of the curriculum’s impact on scenario-based decision quality. Self-efficacy was the stronger mediator, accounting for approximately 28% of the total effect compared with 13% for future-orientation. Although the treatment → CFC (*a*) path was only marginally significant in the difference-in-differences model (*p* = 0.084), the CFC indirect effect remains statistically significant because mediation depends on the product of the *a* and *b* paths rather than on the *a* path alone; this indirect estimate should nonetheless be read with corresponding caution. When each path of the model is re-estimated under the enumerated wild cluster bootstrap, however, every path sits at the structural floor (both *a* paths, both *b* paths, and *c*′: all WCB *p* = 0.125), so the mediation pattern receives no support at the unit of randomization. The Imai et al. (2010b) sensitivity analysis reinforces this caution: the FSES indirect effect is nullified at a mediator–outcome error correlation of *ρ* = 0.147 (equivalent to an unobserved confounder explaining about 2.2% of residual variance in both mediator and outcome), and the CFC indirect effect at *ρ* = 0.051, indicating fragile estimates. The parallel-pathway pattern should therefore be read as hypothesis-generating.

**Table 3:** Bootstrap multiple-mediator analysis of treatment → behavioral decision quality.

Path	Estimate	Boot SE	95% CI	Significant
Indirect via FSES	*0.038*	0.009	[0.022, 0.056]	✓
Indirect via CFC	*0.018*	0.006	[0.008, 0.030]	✓
Total indirect	*0.056*	0.010	[0.037, 0.078]	✓
Direct (c′)	*0.079*	0.026	[0.026, 0.129]	✓
Total	*0.135*	0.024	[0.086, 0.182]	✓

2,000 bootstrap resamples; individual-level inference. Mediator models adjust for the mediator’s own baseline and gender; the outcome model adjusts for baseline BDQ and gender. Clustering cannot be modeled in the indirect-effect CIs with four clusters; path-level wild-cluster-bootstrap results and the mediator–outcome confounding sensitivity analysis are reported in the text. CIs are percentile intervals. ✓ = 95% CI excludes zero under individual-level inference only. Italicized estimates indicate that the 95% bootstrap confidence interval excludes zero under individual-level inference only.

**Figure 3:**
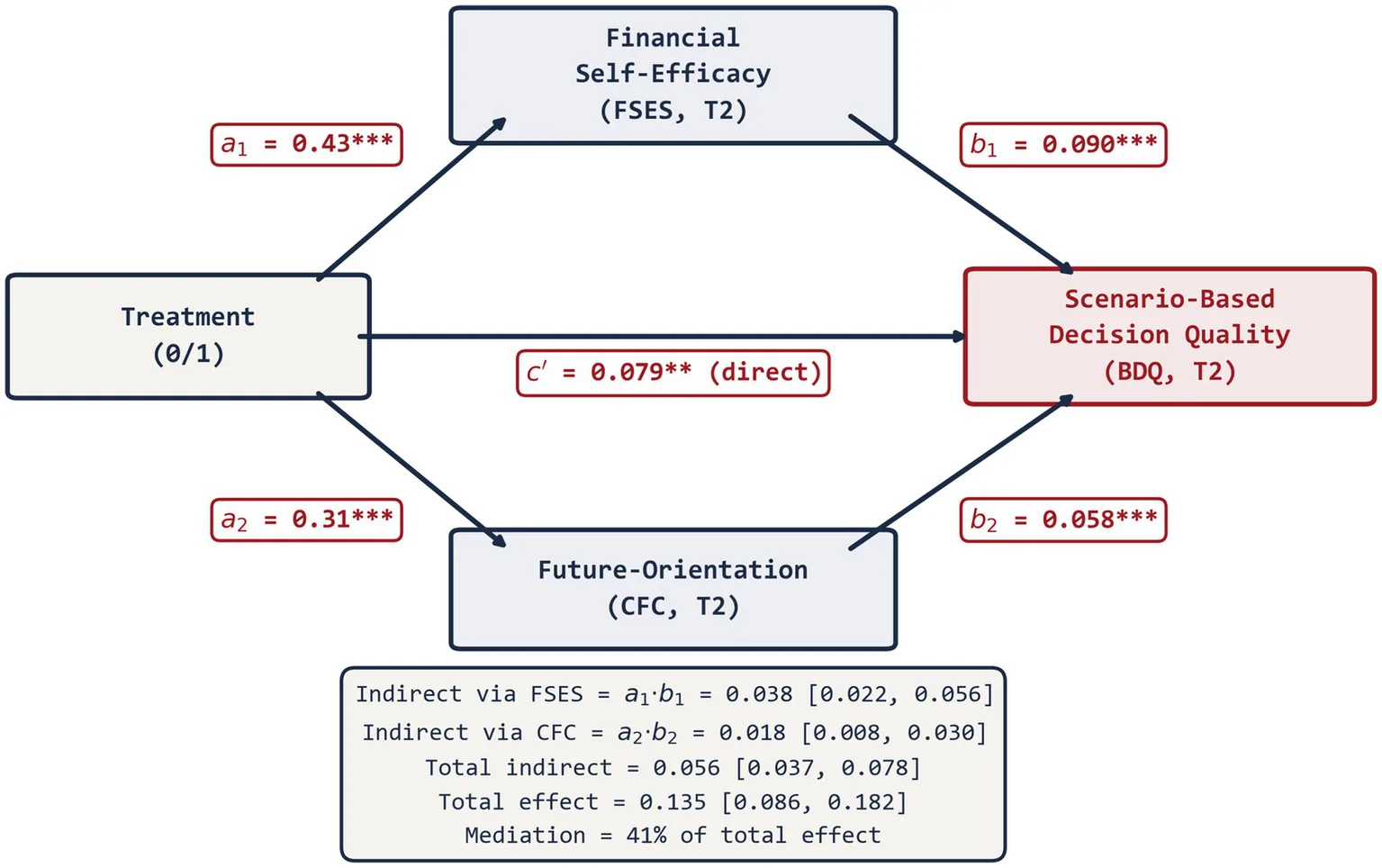
Multiple-mediation model with self-efficacy and future-orientation (CFC) as parallel mediators (exploratory, individual-level) **p<0.01; ***p<0.001 (individual-level inference). Unstandardized coefficients from the exploratory mediation models (each mediator adjusted for its own baseline; outcome model adjusted for baseline BDG and gender); percentile bootstrap 95% CIs in brackets (2,000 resamples). Under the enumerated wild cluster bootstrap all paths sit at the structural floor (*p* =0.125; see section 4.6); the treatment-CFC path was marginal in the difference-in-differences specification (*p* =0.084; Table 2).

The mediation structure is visualized in Figure 3.

### 4.7 Sensitivity and heterogeneity analyses

Sensitivity analyses: per-protocol analysis (treatment subset attending ≥ 6/8 sessions) produced point estimates within 12% of the primary as-randomized estimates and did not change conclusions (e.g., FSES per-protocol *d* = 0.55 vs. as-randomized *d* = 0.51). Inverse-probability-weighted analyses for attrition produced essentially identical results (all *p*s within ±0.005 of primary). Winsorization at the 1st/99th percentiles likewise left effects substantively unchanged. ANCOVA replacing DID produced equivalent inferences. These convergent results support the robustness of our primary findings under individual-level inference; however, as discussed in Section 4.4, all main estimates fail to attain conventional 0.05 significance under wild cluster bootstrap due to the small-J constraint—a limitation we address explicitly in Section 5.3.

Heterogeneity analyses (exploratory): no robust heterogeneity by baseline literacy was detected. The treatment × below-median-baseline interaction was non-significant for both FLS (*p* = 0.253) and FSES (*p* = 0.188), and the direction of the subgroup difference was not stable across reasonable specifications (median-split boundary and estimator choices). Consistent with regression to the mean, below-median control students improved by +0.68 points on FLS from T1 to T2 while above-median control students declined by 0.68 in the absence of any intervention, underscoring why subgroup contrasts defined on noisy baseline scores must be interpreted with care. We note that a low-baseline advantage would in any case have run counter to meta-analytic evidence that financial education is, if anything, less effective for low-income populations (Kaiser and Menkhoff, 2017). No substantive gender × treatment or family income × treatment interactions were detected.

## 5 Discussion

This cluster-randomized controlled trial evaluated a brief, four-week financial literacy curriculum among Chinese vocational college students. Under cluster-respecting inference—the appropriate basis for causal claims with class-level randomization—no outcome attained conventional significance: all immediate treatment estimates sit at the structural floor of the enumerated wild cluster bootstrap (*p* = 0.125 with four clusters), and the trial cannot reject the null at the unit of randomization. Individual-level estimates suggested improvements in financial knowledge (*d* = 0.37), self-efficacy (*d* = 0.51), and scenario-based decision quality (*d* = 0.48), and exploratory mediation analysis suggested that approximately 41% of the individual-level effect on decision quality may operate through financial self-efficacy and future-orientation jointly—a pattern that connects two previously separate mechanism-focused literatures and provides concrete hypotheses for adequately clustered trials.

The magnitude of the individual-level estimates warrants suspicion rather than confidence. Effects two to five times larger than the pooled estimates from 76 randomized experiments (Kaiser et al., 2022: roughly *d* = 0.20 for knowledge and *d* = 0.10 for behavior), arising from a single four-cluster study, are more consistent with bias than with a superior curriculum. Three mechanisms are plausible. First, expectancy and demand effects: the sole instructor was the unblinded principal investigator, and four of six outcomes are self-report. Second, outcome–curriculum proximity: the BDQ scenarios exercise decision types the curriculum rehearsed, unlike the field behaviors aggregated in the meta-analysis. Third, small-study effects: single-site, few-cluster trials are the stratum in which inflated effects are most common. We therefore regard *d* = 0.48 as an upper bound; unbiased, adequately clustered replication is likely to yield smaller effects.

### 5.1 Theoretical contributions

Three contributions extend the financial literacy education literature.

First, to our knowledge we provide the first integrated mediator model combining self-efficacy and future-orientation within a single financial-education RCT. The exploratory, individual-level pattern—both pathways showing indirect effects—is consistent with the hypothesis that financial education is not solely a “knowledge transmission” intervention but also reshapes psychological resources and future-orientation. Given the concurrent measurement of mediators and outcome, the observational mediator–outcome paths, and the absence of cluster-level support (Section 4.6), this dual-pathway account is a hypothesis for confirmation, not an established causal decomposition; it nonetheless clarifies why knowledge-only outcome measures may understate what curricula change. The 41% mediation share also implies that 59% of the total effect operates through pathways outside our two measured mediators—a substantial residual that deserves direct future investigation (see Section 5.4 for candidate mechanisms). Practically, this suggests that future RCTs should report decomposed indirect effects across multiple mediators rather than relying solely on knowledge-based proxies, and should systematically expand the mediator set to identify the unmeasured 59%.

H3 partial support and its implications. H3 received only partial confirmation: future-orientation (CFC) shifted in the predicted direction at marginal significance (*p* = 0.084); risk attitude (DOSPERT) and debt attitude (DAS) showed point estimates in the predicted direction but did not reach significance at T2 or T3. Two interpretations are plausible. First, our four-week dosage may be insufficient to shift deeply ingrained risk and debt attitudes, which prior literature has shown require sustained or repeated intervention to budge (consistent with Iterbeke et al., 2020, who reported preference shifts only after 6 months); the marginal CFC effect, in contrast, suggests future-orientation is more responsive to brief curricula. Second, the variability of DOSPERT-Financial in young vocational samples may be censored relative to working-age populations, yielding lower statistical power. We caution against interpreting the null DOSPERT and DAS findings as definitive evidence of no effect; rather, they likely reflect dosage-power constraints that future longer or repeated-dose interventions should test directly.

Second, by sampling Chinese vocational students, we extend the evidence base to a population representing over 16 million tertiary enrollees with documented low baseline financial literacy. The directional consistency of our effects with international RCTs in higher-income settings (Bover et al., 2026; Sconti et al., 2024) supports the generalizability of the financial education paradigm. We found no robust heterogeneity by baseline literacy (Section 4.7), so we draw no subgroup-targeting implications.

Third, the descriptive durability contrast between T2 and T3 documented here joins the small but growing body of longer-horizon follow-up evidence in financial education RCTs (Frisancho, 2023; Bruhn et al., 2016). The self-efficacy trend (directional but non-significant at T3) in contrast with the full decay of knowledge estimates is consistent with the hypothesis that psychological gains may be more durable than purely cognitive ones—as self-efficacy theory predicts (Bandura, 1997), since perceived competence becomes self-reinforcing through subsequent behavioral feedback. If confirmed, this pattern would have direct curricular implications: brief curricula should explicitly target self-efficacy formation rather than treating it as incidental.

### 5.2 Practical and policy implications

For curriculum designers in Chinese vocational education, our findings imply three actionable conclusions:

- Brief curricula (eight 90-min sessions; 12 total hours) may achieve meaningful change in decision-making tendencies—pending confirmation in adequately clustered trials, suggesting potentially cost-effective scalability to the broader Chinese vocational system without requiring extensive curricular displacement.
- Explicit attention to self-efficacy strengthening within the curriculum (rather than only knowledge delivery) is a promising curriculum component to test in adequately clustered future trials, rather than an established amplifier of impact. In our exploratory mediation analysis, the Week 4 module—which combined intertemporal-choice instruction with personal six-month financial planning—was associated with the largest mediator-channeled estimates.
- Booster sessions or refresher curricula at 3–6-month intervals may be needed to maintain knowledge gains beyond the immediate post-intervention window. Future implementations could systematically test booster designs against single-dose curricula.

Policy actors implementing China’s Ministry of Education and People’s Bank of China Financial Literacy Education Standard Framework (2018) may particularly benefit from these guidelines for the vocational education subsector, currently underserved by tailored curricula.

### 5.3 Limitations

This study has eight limitations that qualify our conclusions and directly inform avenues for future work.

1. Small cluster count (*J* = 4) constrains cluster-level inference. Although individual-level OLS with HC3 robust SE produced treatment effects with *p* < 0.05 for FLS, FSES, and BDQ, the enumerated wild cluster bootstrap—our primary cluster-respecting inference—produced *p* values uniformly at its structural floor (2/16 = 0.125) for all immediate effects. This is a structural feature of any RCT with so few clusters and is not a substantive null result; rather, it reflects the limited information content of cluster-level variation when only four classes are observed. We have reported both inference frameworks transparently. Future replications should aim for at least 8–12 clusters per arm, which would enable conventional cluster-level significance assessment and substantially strengthen causal inference. Our findings should be interpreted as effect-size estimates supported only under individual-level inference; cluster-level inference is bounded by design, and causal claims at the unit of randomization are not supported. Exact cluster-level randomization inference reinforced this reading: the observed allocation produced the largest treatment effect among all six possible class-to-arm assignments for every immediate (T2) outcome, yet—because randomization occurred over only four clusters—the two-sided randomization *p* value has a structural floor of 1/3 (all six T2 effects: *p* = 0.333), so conventional cluster-level significance is unattainable by design rather than by absence of effect.
2. Concurrent measurement of mediators and outcome at T2 limits causal mediation interpretation. FSES, CFC, and BDQ were all measured immediately post-intervention, leaving open the possibility of reverse causation (e.g., a high-quality decision experience increasing self-efficacy rather than self-efficacy enabling the decision). While our analysis adjusts for baseline BDQ_T1 and aligns with the predominant cross-sectional mediation tradition in financial education research (cf. Çera et al., 2021), strict causal mediation requires temporal precedence of M before Y. In addition, random assignment identifies only the treatment → mediator paths; the mediator → outcome paths remain observational and vulnerable to unmeasured confounding. The sensitivity analysis reported in Section 4.6 quantifies this vulnerability (indirect effects nullified at *ρ* = 0.147 for FSES and ρ = 0.051 for CFC), and we accordingly treat H5 as exploratory. Future designs should measure mediators during weeks 2–3 of the intervention—before the T2 outcome assessment—to address this temporal concern directly.
3. Single-instructor design precludes instructor blinding. The PI delivered both treatment and active-control sessions, ensuring instructor-quality consistency but preventing instructor-blinding to study hypothesis. While our active-control design and survey administration by RA mitigate gross expectancy effects, we cannot fully rule out subtle differential investment in the two arms. Expectancy and demand effects therefore remain uncontrolled for all self-report outcomes, and the between-arm difference in perceived course quality (4.16 vs. 3.78) is equally consistent with such effects; this concern compounds the effect-size caution discussed at the opening of this section. Future replications should employ separate instructors per arm with masking to study hypothesis.
4. Single-site sampling. The trial was conducted in a single vocational college in one city, limiting external generalizability. Multi-site replication across regions, urban/rural divides, and college types is needed.
5. The outcome index relies on simulated scenarios. The BDQ index, while showing meaningful convergent validity with self-reported planning behaviors at baseline (mean *r* = 0.33), reflects decision-making tendencies in simulated scenarios rather than actual financial behavior in the field. Future studies should incorporate administrative data—e.g., actual bank transactions, savings records, credit applications—to strengthen behavioral inference.
6. FSES Chinese translation needs formal psychometric validation. While translate-back-translate procedures and pilot reliability assessment (*N* = 40, *α* = 0.81) provide preliminary support, formal CFA validation in a large Chinese vocational sample remains a needed step before broad deployment. Relatedly, the FLS showed modest reliability (KR-20 = 0.68), which attenuates and destabilizes the literacy-outcome estimates specifically; both measurement limitations qualify the corresponding conclusions wherever they appear.
7. Six-month follow-up may be insufficient for durable financial-behavior assessment. While our T3 measurement contributes to the small but growing long-term follow-up literature, ideal financial-behavior research would track participants over 2–5 years to assess durable change in cumulative outcomes. A two-year follow-up extension is planned.
8. Substantial residual direct effect (59% of total) indicates unmeasured mechanisms. Although both FSES and CFC showed indirect effects under individual-level inference, 59% of the total treatment effect on BDQ operates outside these two pathways. Candidates for future investigation include mental accounting (Thaler, 1985), financial socialization (Shim et al., 2009), and peer-effect channels Bursztyn et al. (2014) through which financial literacy training may propagate within social networks.

### 5.4 Future research directions

Building on the present design, two directions take priority because they address this trial’s binding limitations directly:

1. Larger-cluster, multi-site replication: a multi-site cluster RCT with at least 12 clusters per arm would enable conventional cluster-level inference and address external validity concerns simultaneously. Co-coordination across 3–4 Chinese vocational colleges in different regions would also test heterogeneity of effects across socioeconomic and regional contexts.
2. Temporal-precedence mediation design: by measuring mediators during the intervention window (weeks 2–3) rather than at T2 endpoint, future studies can establish stronger causal-mediation evidence than the present cross-sectional mediation framework allows.

Secondary directions include booster-dose comparisons to map the dose–response curve of the partial decay we documented, linkage to administrative or app-based behavioral data to convert scenario-based indices into observed behavior, and expanded mediator sets (e.g., mental accounting, financial socialization, peer effects) to probe the unexplained 59% of the total effect.

## 6 Conclusion

A four-week, eight-session financial literacy curriculum was associated with individual-level improvements in financial knowledge, self-efficacy, and scenario-based decision-making among Chinese vocational college students; under cluster-respecting inference, however, no effect attained conventional significance, and cluster-level significance is not attained in this trial. Exploratory mediation analysis suggested parallel psychological pathways—self-efficacy and future-orientation—jointly accounting for 41% of the individual-level effect, a hypothesis-generating pattern whose fragility to mediator–outcome confounding we quantify. Knowledge estimates fully attenuated by 6 months while self-efficacy showed a directional but non-significant trend toward persistence. These findings provide effect-size estimates, measurement groundwork, and mechanism hypotheses for adequately clustered confirmatory trials in an underserved Chinese vocational population.

## Data Availability

The datasets presented in this study can be found in online repositories. The names of the repository/repositories and accession number(s) can be found at: the de-identified three-wave dataset and complete analysis code supporting the conclusions of this article are publicly available in the Open Science Framework repository at https://osf.io/ep7v4 (DOI: 10.17605/OSF.IO/EP7V4).
