# Supplementary material for: Psychological mechanisms linking a financial-literacy curriculum to scenario-based financial decision quality: a cluster randomized trial of financial self-efficacy and future-orientation in Chinese vocational students
Source: Front Psychol. 2026 Aug 10;17:1906773. doi: 10.3389/fpsyg.2026.1906773 (PMC13500269; doi:10.3389/fpsyg.2026.1906773)
Supplement: Supplementary file 1 [file Supplementary_file_1.DOCX]

# Supplementary Materials

# S1. Four-Week Financial-Literacy Curriculum (Intervention Protocol)

**Purpose.** Intervention delivered to the treatment arm; the active-control arm received an equivalent block of their regular curriculum.

**Dose.** 4 weeks × 1 session/week × 2 class periods/session (90 min) = **8 sessions / 360 minutes** (a lightweight adaptation of the ~10-session framework of Bover, Hospido, & Villanueva for the Spanish high-school RCT, scaled to Chinese vocational-college timetabling).

**Participants.** Vocational-college students in **non-finance/non-accounting majors** (to avoid overlap with prior disciplinary knowledge).

## Overall Design Logic

The four weeks map onto the **four classes of psychological outcome measured in the study**: each week's content corresponds to one instrument, facilitating identification of intervention effects.

| **Week** | **Theme** | **Linked instrument** | **Theoretical basis** |
| --- | --- | --- | --- |
| W1 | Financial-literacy fundamentals (the "Big Three") | **FLS** (financial knowledge) | Behavioral finance — the knowledge basis of rational decision-making |
| W2 | Risk and decision-making | **DOSPERT** (risk attitude) | Prospect theory / risk perception |
| W3 | Debt and credit | **DAS** (debt attitude) | Mental accounting / self-control |
| W4 | Intertemporal choice and behavioral biases | **CFC** (time preference) + **FSES** (self-efficacy) | Intertemporal choice / self-efficacy theory |

**Design principle.** Each week adds one knowledge domain and reinforces the "knowledge → confidence → behavior" translation. Each week ends with a **real-world transfer task** (e.g., draft next month's budget; simulate a small financial decision).

## W1 · Financial-Literacy Fundamentals (the Big Three + inflation)

**Learning objectives** (by the end, students can):

1. 1.Explain **compound interest** (the interaction of rate and time) and solve three compounding problems.
2. 2.Distinguish **nominal vs. real interest rates** and explain inflation's effect on the purchasing power of savings.
3. 3.Understand the logic of **risk diversification** (a quantitative account of "don't put all your eggs in one basket").
4. 4.Answer all of Lusardi and Mitchell's "Big Three" financial-literacy items correctly.

**Session plan (90 min).**

- 0–15 min — **Warm-up:** students receive ¥100 in play money and choose among "all to savings / all to a fund / split evenly," record their choice, and are immediately shown the divergent 5-year outcomes (creating cognitive conflict).
- 15–50 min — **Core instruction:** compound-interest formula A = P(1 + r)^n with a linear-vs-exponential plot; the 10-year erosion of ¥10,000 in cash at 3% annual inflation; variance comparison of holding 1 vs. 10 vs. 100 stocks.
- 50–75 min — **Group discussion** (groups of four): "You have just graduated and earn ¥5,000/month — what is your financial advice?"
- 75–90 min — **In-class Big Three quiz** with immediate feedback.

**Assignment (due within 1 week):** a 300-character reflection, "My compound-interest vision of my finances over the next 5 years."

**Key case material:** real-effective-interest comparison of "12 interest-free installments" vs. "discount for full payment"; China's CPI trend over the past decade mapped to purchasing power.

## W2 · Risk and Decision-Making (DOSPERT five domains + risk-attitude formation)

**Learning objectives.**

1. 5.Distinguish **risk perception** from **risk preference** (rarely separated in textbooks).
2. 6.Understand DOSPERT's five domains (financial, health, recreational, ethical, social) — why "daring to bungee-jump" and "daring to trade stocks" are distinct.
3. 7.Identify three **risk-decision biases**: loss aversion, overconfidence, reference-point effects (an introduction to Kahneman and Tversky).
4. 8.Complete a simulated portfolio-allocation exercise.

**Session plan.**

- 0–10 min — **Warm-up:** complete a short DOSPERT-style form (presented as a teaching tool, not a research instrument), collected anonymously in class.
- 10–45 min — **Instruction:** the core prospect-theory figure (value function + decision weights) and DOSPERT's five domains.
- 45–70 min — **Group activity:** with ¥10,000 in play money and five products of differing risk (demand deposit, bond fund, balanced fund, equities, cryptocurrency), each student allocates and justifies their choice.
- 70–90 min — **Reflective discussion:** why do allocations differ — risk perception or risk preference?

**Assignment:** observe three real decisions over one week (not limited to money) and analyze whether you weighted gains or feared losses.

**Key case material:** panic-selling among retail investors (loss aversion); the psychology of group-buying nudges (overconfidence + reference points); recognizing risk signals in scams common among vocational students (fake part-time "click-farming," predatory online lending).

## W3 · Debt and Credit (DAS dimensions + mental accounting)

**Learning objectives.**

1. 9.Distinguish **constructive debt** (education, housing) from **consumer debt** (revolving credit-card interest, online loans).
2. 10.Compute the true annualized cost of revolving credit-card interest (a deliberately effortful calculation — central to behavior change).
3. 11.Understand **mental accounting** — why spending via consumer-credit apps feels "less painful" than spending cash.
4. 12.Learn to check one's personal credit record and recognize its consequences.
5. 13.Complete a debt-attitude self-check.

**Session plan.**

- 0–15 min — **Hook:** a real case of a student repaying a ¥5,000 credit-card bill over 24 installments and the total excess paid (students guess first, then the figure is revealed).
- 15–50 min — **Instruction:** decomposing the interest of credit cards / consumer-credit apps / online loans (APR vs. effective annual rate); mental-accounting theory (Thaler) and the "painlessness" of digital payment; how to obtain a personal credit report (People's Bank of China Credit Reference Center).
- 50–75 min — **Group activity:** design a 6-month repayment plan for a "student earning ¥5,000/month with ¥3,000 of debt."
- 75–90 min — **Self-assessment:** complete a short DAS and self-identify one's type (disclosure not required).

**Assignment:** (i) obtain your own credit report (guidance provided); (ii) a 200-character reflection, "My attitude toward debt."

**Key case material:** news reports on predatory campus lending; a cost comparison of installment vs. full payment for an appliance; a "how much did interest-free credit cost me this year" self-audit.

## W4 · Intertemporal Choice, Behavioral Biases, and Self-Efficacy

**Learning objectives.**

1. 14.Understand **intertemporal choice** — why many prefer "¥100 now" over "¥110 in a year."
2. 15.Master three tools to counter **present bias**: automatic transfers, a "21-day savings challenge," and psychological pre-commitment.
3. 16.Identify five behavioral-finance biases: status-quo bias, anchoring, mental accounting, overconfidence, loss aversion.
4. 17.Strengthen the belief that "I can manage my own money" (financial self-efficacy; a key mediator).
5. 18.Complete a personal six-month financial plan.

**Session plan.**

- 0–15 min — **Classic demonstrations:** the Strathman CFC items in class and the "marshmallow test" as a case study.
- 15–45 min — **Instruction:** hyperbolic discounting (why we favor the present); a quick guide to five behavioral biases with in-class identification practice; self-efficacy theory (Bandura) and the cognitive reframing of "I can't" into "I can."
- 45–75 min — **Applied task:** each student drafts a six-month financial plan with a concrete budget, savings goal, and risk exposure.
- 75–90 min — **Closing:** each student shares one sentence on "the biggest change in how I see my finances after four weeks." (This activity is intended to consolidate students' sense of capability.)

**Assignment:** address the six-month financial plan to one's future self (a sealed letter, opened at the six-month behavioral follow-up).

## Appendix A. Control of Differences from the Active-Control Arm

| **Dimension** | **Treatment arm** | **Active-control arm** |
| --- | --- | --- |
| Dose | 8 sessions of financial-literacy curriculum | 8 sessions of an **equivalent block** of their regular curriculum (e.g., relevant chapters of a Career-Development module) |
| Instructor | The instructor | The same (or an equally qualified) instructor — same instructor preferred to control for instructor effects |
| Measurement | T1 (baseline) / T2 (immediate) / T3 (6 months) | Same T1 / T2 / T3 |
| Consent | Informed consent + voluntary participation | Informed consent + voluntary participation |

**Ethical safeguard.** After the study, control-arm students received the same financial-literacy materials (slides + handouts) free of charge, in line with the IRB's equity provisions.

## Appendix B. Teaching-Materials Checklist

| **Item** | **Quantity** | **Responsible** |
| --- | --- | --- |
| Weekly slide decks | 4 sets | Instructor (developed), co-author (reviewed) |
| In-class activity cards (W1 play money / W2 portfolio / W3 case cards) | 1 set/week | Instructor |
| Student handouts | 100 copies × 4 weeks | Instructor |
| Pre/post scale forms or links | See Supplement B | Instructor |
| Big Three quiz items (Chinese version of Lusardi & Mitchell) | 1 set | Instructor (translated), co-author (reviewed) |

## Appendix C. Course-Evaluation Form (manipulation check, reported in the Methods)

At the end of the final session, students completed a 5-item, 5-point Likert evaluation (1 = strongly disagree, 5 = strongly agree):

1. 19.I learned practical financial knowledge from these four weeks.
2. 20.The content is likely to affect my financial decisions.
3. 21.The instruction was clear and the cases were relevant to daily life.
4. 22.The discussions gave me new ways of thinking about money.
5. 23.I will apply these methods in my own life.

A mean score ≥ 4.0 was defined a priori as the minimum threshold for "successful implementation" and is reported in the Methods as a manipulation check.

# S2. Integrated Questionnaire (Baseline / Post-Test / 6-Month Follow-Up)

**Purpose.** The three-wave measurement instrument for the RCT (administered in Chinese via an online survey platform).

**Estimated completion time.** Baseline 12–15 min; post-test 12–15 min; 6-month follow-up 10–12 min.

**Total items.** ~75 (including demographics).

***Note on instruments.*** *The scales were administered under their academic-use terms; the items below are the authors’ own Chinese-language adaptations of the cited source scales, not verbatim reproductions of the original instruments. Because Lown's (2011) Financial Self-Efficacy Scale has no official Chinese version, the Chinese wording used here is a preliminary translation; before formal use it underwent translate–back-translate procedures and a small-sample (N = 40) reliability pilot (see Limitations in the main text). The English wording shown is a back-translation of the adapted Chinese items, provided for the reader; each instrument is attributed to its source below.*

## Part 1 · Informed Consent (T1 only)

*We are conducting an educational study on "the effect of financial-literacy education on students' financial decision-making." Participation involves completing this questionnaire (~12 min), attending a 4-week course, completing a follow-up questionnaire at the end of the course, and completing one further questionnaire 6 months later.*

*Your rights: participation is entirely voluntary and you may withdraw at any time without affecting any grade; all responses are processed anonymously and results are reported only as group-level data; data are held by the research team, and no identifying personal information appears in any publication.*

*☐ I have read the above and agree to participate.*

## Part 2 · Demographics (full at T1; brief at T2/T3)

| **No.** | **Item** | **Options / format** | **Wave** |
| --- | --- | --- | --- |
| D1 | **Last 4 digits of student ID + birth month** (for matching; identity not visible to researchers) | 6-digit code | T1/T2/T3 |
| D2 | Gender | Male / Female / Other / Prefer not to say | T1 only |
| D3 | Year and month of birth | yyyy-mm | T1 only |
| D4 | Class | Dropdown | T1 only |
| D5 | Major | Text | T1 only |
| D6 | Parents' highest education | ≤ Junior high / Senior high / Junior college / Bachelor's / Master's–Doctoral | T1 only |
| D7 | Approximate monthly household income (CNY) | <3,000 / 3,000–5,000 / 5,000–10,000 / 10,000–20,000 / >20,000 / Unsure | T1 only |
| D8 | Do you have part-time income? | Yes / No | T1/T3 |
| D9 | Do you hold any consumer-credit tools (credit card, consumer-credit apps)? | Yes / No | T1/T3 |
| D10 | Have you used campus or online loans? | Yes / No / Prefer not to say | T1/T3 |

D1 rationale: the last four ID digits + birth month form an anonymous matching code that links the three waves without collecting the full student ID, consistent with IRB data-minimization principles.

## Part 3 · Financial-Literacy Knowledge (FLS; T1/T2/T3)

*Adapted from Lusardi & Mitchell (2014) "Big Three" plus extension items. (✓ marks the correct answer.)*

**F1 (compounding).** Suppose you have ¥100 in a savings account earning 2% per year. After **5 years**, how much would be in the account (no withdrawals)?

A. More than ¥102 ✓ B. Exactly ¥102 C. Less than ¥102 D. Don't know

**F2 (inflation).** Suppose your account earns 1% per year and annual inflation is 2%. After **1 year**, what you can buy with the money would be…

A. More B. The same C. **Less** ✓ D. Don't know

**F3 (risk diversification).** "Buying a single company's stock usually provides a safer return than buying a stock mutual fund." True or false?

A. True B. **False** ✓ C. Don't know

**F4 (interest-rate sensitivity).** If market interest rates fall, bond prices usually…

A. **Rise** ✓ B. Fall C. Stay the same D. Don't know

**F5 (applied knowledge).** When you pay only the minimum on a credit card rather than the full balance, the remaining balance is charged interest…

A. No interest B. **On the outstanding principal (including partly repaid principal)** ✓ C. Recalculated at the next due date D. Don't know

Scoring: 1 point per correct answer (0–5). 0–2 low, 3 medium, 4–5 high. At T2 the item order is shuffled and wording paraphrased equivalently to limit memory effects.

## Part 4 · Risk Attitude (DOSPERT-Financial subscale; T1/T2/T3)

*Adapted from the DOSPERT financial-risk subscale (Weber, Blais, & Betz, 2002; revised open-access version: Blais & Weber, 2006, Judgment and Decision Making, 1, 33-47), 6 items.*

Rate how **likely you would be to do** each of the following (1 = extremely unlikely, 7 = extremely likely):

| **No.** | **Item** |
| --- | --- |
| R1 | Invest one month's income in a volatile, potentially high-return stock |
| R2 | Put all your savings into a single emerging-industry fund |
| R3 | Borrow money for an investment with a 50% chance of profit |
| R4 | Invest 10% of annual income in a moderate-risk investment |
| R5 | Put 5% of income into a stable blue-chip stock |
| R6 | Keep savings in a fixed-rate bank deposit (**reverse-scored**) |

Scoring: 1–7 Likert, R6 reverse-scored; higher = greater financial risk preference.

## Part 5 · Debt Attitude (DAS, short form; T1/T2/T3)

*Adapted from Davies & Lea (1995) and Norvilitis et al. (2003), short form, 8 items.*

Indicate your agreement (1 = strongly disagree, 5 = strongly agree):

| **No.** | **Item** |
| --- | --- |
| DA1 | Using credit lets me buy things I cannot currently afford |
| DA2 | Borrowing to consume makes me uneasy (**reverse**) |
| DA3 | Overdrawing now and repaying gradually is a reasonable way to live |
| DA4 | I would rather buy less than be in debt (**reverse**) |
| DA5 | My classmates all use consumer credit, so I do too |
| DA6 | Credit tools let me enjoy life more |
| DA7 | I carefully calculate the interest cost of each loan (**reverse**) |
| DA8 | A good credit record is important for my future (**reverse**) |

Scoring: DA2/DA4/DA7/DA8 reverse-scored; higher = more permissive/risky debt attitude. Two sub-dimensions can be derived: impulsive debt attitude (DA1/3/5/6) and rational-management attitude (DA2/4/7/8, after reversal).

## Part 6 · Time Preference / Intertemporal Choice (CFC, short form; T1/T2/T3)

*Adapted from Strathman, Gleicher, Boninger, & Edwards (1994), Consideration of Future Consequences Scale, short form, 7 items.*

Rate how well each statement describes you (1 = not at all like me, 5 = very much like me):

| **No.** | **Item** |
| --- | --- |
| C1 | When I make decisions, I think about how they may affect me many years ahead |
| C2 | I often consider only the immediate benefit when deciding (**reverse**) |
| C3 | I believe my present choices will affect me years from now |
| C4 | I would rather be comfortable now than prepare for 5 years ahead (**reverse**) |
| C5 | I will sacrifice present comfort for long-term future gains |
| C6 | I weight long-term goals more than immediate outcomes |
| C7 | The future is beyond my control now, so I attend to the present first (**reverse**) |

Scoring: total after reversing reverse-scored items; higher = greater future orientation (CFC-Future).

## Part 7 · Financial Self-Efficacy (FSES; T1/T2/T3) — key mediator

*Adapted from Lown (2011), Financial Self-Efficacy Scale, 6 items.*

*The Chinese wording is a preliminary translation requiring translate–back-translate and an N = 30–50 pilot before formal use.*

Indicate your agreement (1 = not at all like me, 4 = very much like me):

| **No.** | **Item** |
| --- | --- |
| FS1 | It is hard to cope when an unexpected expense arises (**reverse**) |
| FS2 | I find it hard to stick to the budget I set (**reverse**) |
| FS3 | When an unexpected expense occurs, I usually can only borrow to cover it (**reverse**) |
| FS4 | Comparing the merits of several financial products is challenging for me (**reverse**) |
| FS5 | I feel unconfident in my ability to control my current finances effectively (**reverse**) |
| FS6 | Growing my money according to plan is challenging for me (**reverse**) |

Scoring: all items reverse-scored (as in the original scale, where higher = greater self-efficacy). Total 6–24; higher = stronger financial self-efficacy.

## Part 8 · Behavioral Decision-Quality Scenarios (behavioral proxy; T1/T2/T3)

*A core feature: beyond knowledge and attitudes, students make decisions in five realistic scenarios used as a behavioral proxy. (✓ marks the answer scored as higher-quality.)*

**B1 (intertemporal choice).** "¥100 now" or "¥110 in one month"? A. ¥100 now B. ¥110 in one month

**B2 (risk decision).** You have ¥1,000 you do not currently need; you prefer to:

A. Demand deposit (0.3%) B. Money-market fund (1.8%, near risk-free) C. Moderate-risk balanced fund (~5%, ±10%) D. Equities (~8%, ±30%) E. Unsure, ask others

**B3 (debt decision).** Short on money at month-end, a friend recommends a new online-loan app ("7 days interest-free, repay principal at term"):

A. Use it immediately B. Check the rate first, then decide C. **Never use it** ✓ D. It depends

**B4 (consumption decision).** A ¥1,000 item is 10% off today, or 30% off next week but requires a 2-hour wait:

A. Buy today at 10% off B. **Buy next week at 30% off** ✓ C. Do not buy (not needed)

**B5 (planning behavior).** In the past month, did you:

Set a monthly spending budget? (Yes/No) · Track daily spending? (Yes/No) · Have a savings goal? (Yes/No) · Compare two financial products? (Yes/No)

Scoring: a composite "decision-quality score" per an a priori rubric. The 6-month follow-up focuses on B5 as an indicator of sustained behavior change.

## Part 9 · Course Evaluation (T2; treatment arm rates the financial-literacy course, control arm rates the equivalent module)

5-item, 5-point Likert (1 = strongly disagree, 5 = strongly agree):

1. 24.I learned practical financial knowledge from these four weeks.
2. 25.The content is likely to affect my financial decisions.
3. 26.The instruction was clear and the cases were relevant to daily life.
4. 27.The discussions gave me new ways of thinking about money.
5. 28.I will apply these methods in my own life.

Use: manipulation check, reported in the Methods.

## Three-Wave Measurement Structure

| **Wave** | **Timing** | **Parts included** | **Duration** |
| --- | --- | --- | --- |
| **T1 (baseline)** | 1–2 weeks before the intervention | Parts 1–8 (excluding Part 9) | 12–15 min |
| **T2 (immediate post)** | 1–3 days after the intervention | Parts 3–9 (brief demographics) | 12–15 min |
| **T3 (6-month follow-up)** | 6 months after the intervention | Parts 3–8, with added emphasis on behavioral B5 | 10–12 min |

## Data-Collection and Cleaning Rules

1. 29.**Matching:** D1 (last 4 ID digits + birth month) as the anonymous matching code.
2. 30.**Quality control:** flag straight-lining (identical option throughout); flag completion time < 4 min; flag severe contradiction between reverse- and forward-scored items.
3. 31.**Storage:** export from the survey platform to SPSS/Stata; raw IP addresses and social-media accounts are not retained.
4. 32.**Missing data:** within-scale missingness ≤ 5% handled by mean imputation; > 10% scale-missingness excluded for that participant–scale, with multiple-imputation sensitivity analysis as backup.
